# Supplementary figures and images for: Adapting a peer recovery coach-delivered behavioral activation intervention for problematic substance use in a medically underserved community in Baltimore City
Source: PLoS One. 2020 Jan 31;15(1):e0228084. doi: 10.1371/journal.pone.0228084 (PMC6993963; doi:10.1371/journal.pone.0228084)

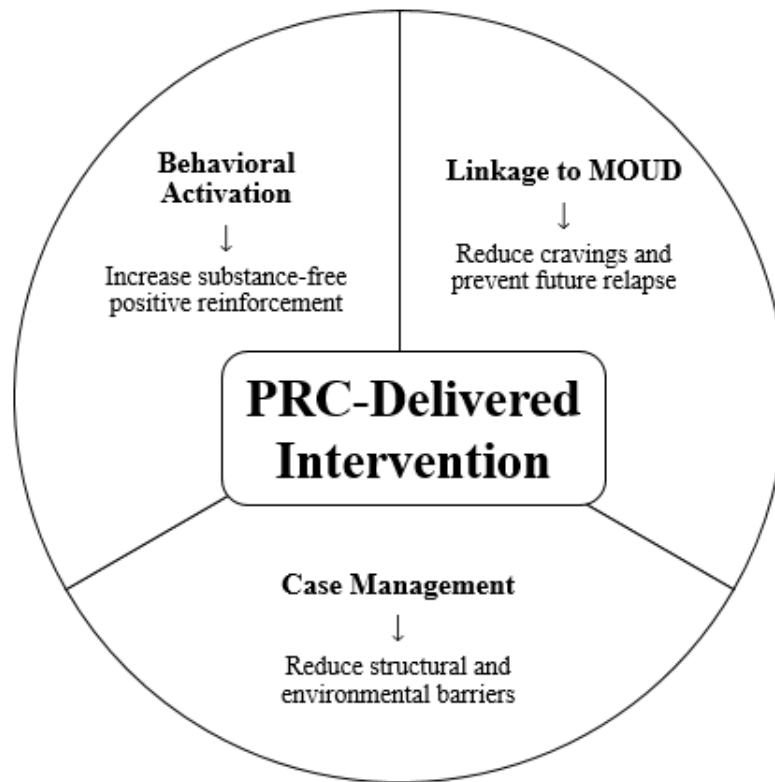

Engage and retain a hard-to-reach population, not otherwise engaged in care

Supplement: S1 Fig — depicts the three intersecting components of the proposed PRC-delivered intervention treatment approach, based upon formative feedback from the KI interviews and FG. To feasibly and effectively engage and retain a hard-to-reach population, and concurrently address structural and psychosocial barriers to treatment, the adapted PRC-delivered intervention developed based on the formative work presented here will incorporate: behavioral activation to increase substance-free positive reinforcement; linkage to MOUD to reduce cravings and prevent future relapse; and case management to address structural and environmental barriers. (PDF) [file pone.0228084.s001.pdf]
