# Supplementary material for: Adapting a peer recovery coach-delivered behavioral activation intervention for problematic substance use in a medically underserved community in Baltimore City
Source: PLoS One. 2020 Jan 31;15(1):e0228084. doi: 10.1371/journal.pone.0228084 (PMC6993963; doi:10.1371/journal.pone.0228084)
Supplement: S1 Appendix — (DOCX) [file pone.0228084.s002.docx]

**POSITIVE EVENTS CHECKLIST**

1. Visiting family
2. Playing soccer/basketball with friends
3. Throwing a Frisbee
4. Going to a friend’s house
5. Going to the movies
6. Going on Facebook and talking to friends
7. Joining a sewing club
8. Going to church/mosque
9. Praying
10. Joining a support group
11. Speaking to a friend on the phone
12. Relaxing in a park or backyard
13. Singing
14. Joining a choir
15. Going for a walk with a friend or partner
16. Playing a game with a child or friend
17. Helping other people
18. Helping the environment by recycling
19. Getting involved in your community
20. Getting a pet
21. Telling jokes and funny stories
22. Bird watching
23. Going to the beach
24. Going on a nature walk
25. Having a cup of tea
26. Starting a collection (shells, pretty stones…)
27. Going on a date
28. Going to a club
29. Relaxing
30. Jogging, walking, running
31. Listening to gospel
32. Reading the bible
33. Reading a book for pleasure
34. Recalling fond memories
35. Reading magazines
36. Lying in the sun
37. Laughing
38. Playing an instrument
39. Meeting new people
40. Eating healthy foods
41. Repairing things around the house
42. Taking care of my plants
43. Starting a small garden
44. Swimming
45. Doodling, drawing, painting
46. Exercising
47. Going to a party
48. Having family get-togethers
49. Having safe sex
50. Doing something spontaneous
51. Doing needlepoint, crocheting, or knitting
52. Joining a social club
53. Flirting/ kissing
54. Making a gift for someone
55. Getting a manicure/pedicure
56. Writing
57. Being with/ playing with children
58. Playing with animals
59. Writing in a journal
60. Writing and sending letters
61. Getting a new haircut
62. Going on a picnic
63. Meditating
64. Doing breathing exercises
65. Seeing and/ or showing photos
66. Dressing up and looking nice
67. Reflecting on how I’ve improved
68. Going to museums
69. Lighting candles
70. Saying “I love you”
71. Thinking about my good qualities
72. Dancing
73. Doing something new
74. Thinking I’m a person who can cope
75. Going to cultural event
